# An Unusual Case of Mixed Respiratory Capillariosis in a Dog

**DOI:** 10.3390/pathogens10020117

**Published:** 2021-01-23

**Authors:** Simone Morelli, Giuseppe Marruchella, Alessandra Passarelli, Anastasia Diakou, Angela Di Cesare, Mariasole Colombo, Antonio Frangipane di Regalbono, Alessandro Frate, Donato Traversa

**Affiliations:** 1 Faculty of Veterinary Medicine, University of Teramo, 64100 Teramo, Italy; gmarruchella@unite.it (G.M.); adicesare@unite.it (A.D.C.); mcolombo@unite.it (M.C.); dtraversa@unite.it (D.T.); 2 Clinica Veterinaria Città di Bari, 70125 Bari, Italy; alessandra_pax@hotmail.com (A.P.); a.frate76@gmail.com (A.F.); 3 Faculty of Veterinary Medicine, Aristotle University of Thessaloniki, 54124 Thessaloniki, Greece; diakou@vet.auth.gr; 4 Department of Animal Medicine, Production and Health, University of Padova, 35020 Legnaro, Italy; antonio.frangipane@unipd.it

**Keywords:** *Capillaria boehmi*, *Capillaria aerophila*, lungs, neurological signs, dogs

## Abstract

Nematodes belonging to the genus *Capillaria* infect a range of domestic and wild animals. *Capillaria aerophila* and *Capillaria boehmi* cause respiratory parasitoses in dogs and wild carnivores, e.g., foxes and mustelids, although they are often overlooked in canine clinical practice. The present report describes an unusual case of a severe and mixed infection by *C. aerophila* and *C. boehmi* in a privately housed dog that showed acute and life-threatening respiratory and neurological signs. Clinic-pathologic and epizootiological implications are described and discussed.

## 1. Introduction

*Capillaria aerophila* (syn. *Eucoleus aerophilus*) and *Capillaria boehmi* (syn. *Eucoleus boehmi*) are capillarid nematodes affecting wild and domestic carnivores. While *C. aerophila* may parasitize different animal species (e.g. dogs, foxes, cats, mustelids) and occasionally humans, *C. boehmi* has a narrow host range and infects mainly wild canids and domestic dogs [1,2,3]. *Capillaria aerophila* and *C. boehmi* inhabit the respiratory system of their hosts, i.e., they live beneath the epithelium of bronchi and trachea and in the nasal cavities and sinuses respectively [3]. Their biology is yet to be elucidated. Animals become infected by *C. aerophila* via the inadvertent ingestion of embryonated eggs from the environment, although earthworms could be somehow (e.g., as facultative intermediate hosts or paratenic hosts) implicated in the life cycle [4,5]. The biological cycle of *C. boehmi* is practically unknown but there is no sound reason to consider it different from that of *C. aerophila* [3].

*Capillaria aerophila* and *C. boehmi* commonly infect wildlife, especially foxes [6,7,8,9,10,11] that are frequently co-infected by both parasites [8]. However, in the last years, they have been recorded in domestic dogs from Europe, North America and Australia, both in monospecific and mixed infections, that are relatively common, and with varying degrees of clinical relevance [11,12,13,14,15,16,17]. Dogs infected by *C. aerophila* and *C. boehmi* could be either subclinically infected or display respiratory clinical signs, in both mixed and monospecific infections [12,15,17]. In particular, pulmonary capillariosis of dogs by *C. aerophila* is a verminous bronchopneumonia characterized by general clinical signs, distress, coughing, sneezing and occasionally airway obstruction [3,12,13]. The nasal infection caused by *C. boehmi* may cause sneezing, itching, mucopurulent nasal discharge, epistaxis, gagging, hypo-/anosmia, that can be severe in the case of heavy parasite burdens [1,17,18,19]. A case of meningoencephalitis with neurological signs caused by the direct migration of *C. boehmi* through the cribriform plate has also been described [20]. To date, there is no evidence of more severe clinical pictures in dogs co-infected by *C. aerophila* and *C. boehmi* compared to monospecific infections.

The factual impact of respiratory capillarioses in canine clinical practice is still underestimated because of the non-specificity of clinical signs and hindrances inherent to the diagnosis [11,15,21]. Given the rise of records of respiratory capillarioses in domestic animals [13,22] and the merit in improving knowledge on these parasitoses, the present report describes an unusual clinical presentation of a life-threatening mixed infection by *C. aerophila* and *C. boehmi* in a dog, and discusses epizootiological and clinic-pathological implications. 

## 2. Case Details

### 2.1. Clinical Case

**Figure 1:**
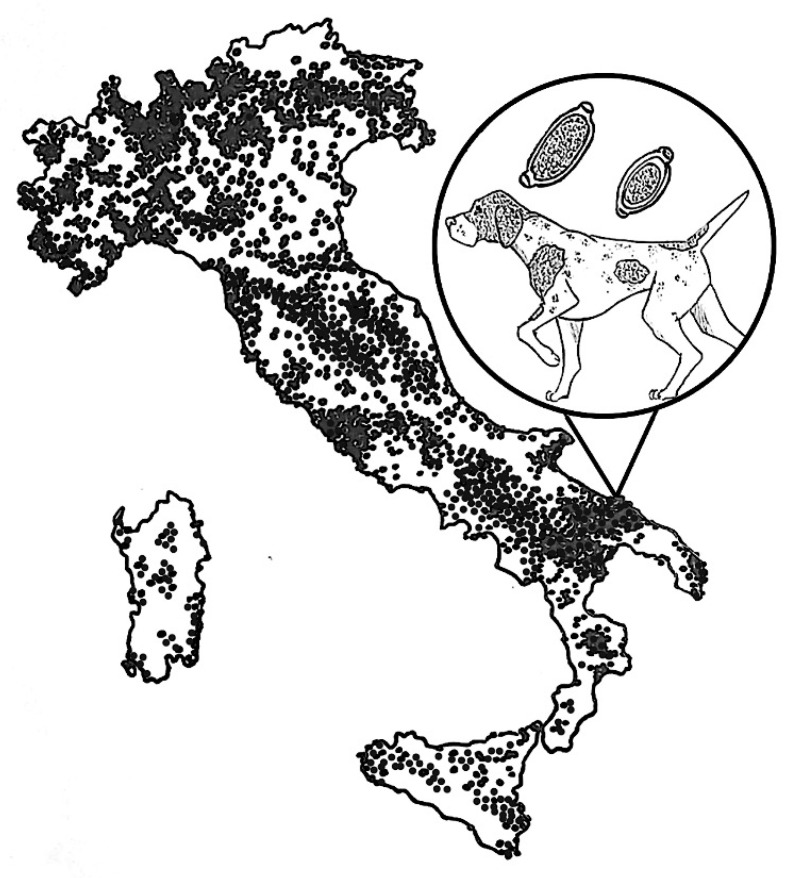
Italy region where the dog of the present case lived. The dots represent the distribution area and density of the red fox (*Vulpes vulpes*) population (https://discovermammals.org/what-you-can-do/learn-more/countries/italy/).

In September 2020, a privately owned 14-year old intact male Pointer, weighing ~27 kg, was presented to a veterinary clinic located in Bari municipality (Apulia region of southern Italy) for an acute onset of coughing, sneezing and dyspnea, alongside intermittent neurological signs, i.e., seizure-like focal and generalized crisis with paddling. The dog lived in a house located in a small fishermen town (i.e., Torre a Mare, 41°05′08.16″ N 16°59′54.6″ E, Figure 1), with a garden, where it had free and continuous access. The animal was kept as a pet, and had travelled only once, in 2016, in a mountainous area of northern Italy (Valtellina, Lombardy region). The dog was not regularly treated with parasiticides nor was subjected to routine copromicroscopic examinations. The physical examination revealed tachypnea (40 breaths/minute), a heart rate of 100 beats per minute, and diffuse pulmonary crackles at the lung auscultation. The examination of the nares and the rostral nasal cavities performed with an otoscope showed hypertrophic and hyperemic nasal mucosae. Thoracic radiology, echocardiography and abdominal ultrasound were performed, and faeces and nasal swabs were collected for parasitological examinations. The blood collected for a complete blood count and serum biochemistry analysis showed values within reference intervals with the exception of a mild left shift (570 band neutrophils/µL; reference interval: 0–300 band neutrophils/µL). A serum aliquot was subjected to the Angio Detect™ test (IDEXX) for the detection of the circulating antigen of *Angiostrongylus vasorum*.

The dog was immediately hospitalized due to critical conditions and treated with diazepam 0.7 mg/kg, prednisolone 1 mg/kg and ceftriaxone 30 mg/kg *sid*. According to the parasitological findings (Section 2.3), the day after the admission, the dog received a spot-on solution containing moxidectin 2.5% (Advocate^®^) but after one further day, the neurological conditions severely and progressively worsened and the owners decided for euthanasia. 

### 2.2. Radiographic and Ultrasound Examinations

**Figure 2:**
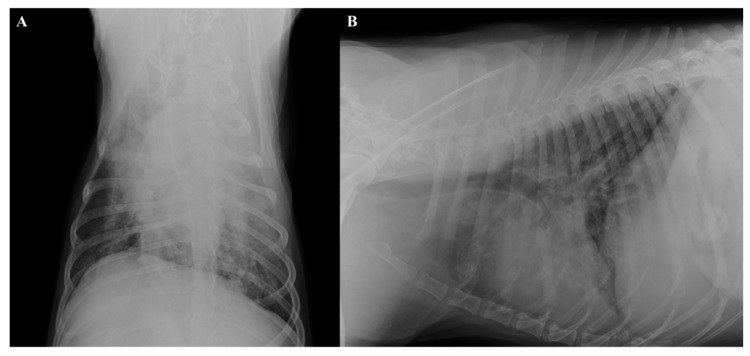
Thoracic radiographs, dorso-ventral (**A**) and latero-lateral (**B**) views. Diffuse bronchiolar and severe interstitial and alveolar patterns and increased sternal contact of the cardiac silhouette (**B**).

Thoracic radiographs showed bronchial pattern along with diffuse and severe interstitial and alveolar patterns (Figure 2A,B). Echocardiography findings were normal. The abdominal ultrasound showed findings compatible with pancreatitis, i.e. pancreas diffusely enlarged with a hyperechoic echo-texture and hyperechoic surrounding mesentery. The prostate was enlarged and showed diffused anechoic areas of heterogeneous size. The liver parenchyma was inhomogeneous, with irregular mixed-echogenic areas within the right lobe. The size of the liver was mildly reduced. 

### 2.3. Parasitological Findings

**Figure 3:**
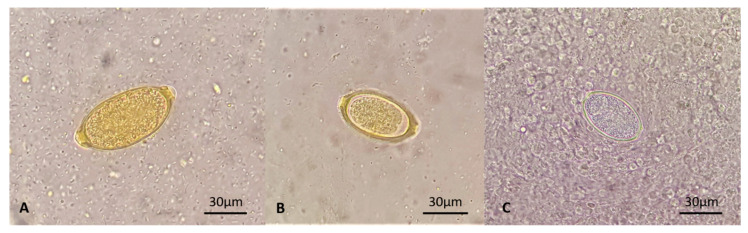
Eggs of *Capillaria aerophila* (**A**), *Capillaria boehmi* (**B**,**C**) retrieved at fecal floatation (**A**,**B**) and the microscopic examination of a nasal swab (**C**).

A classical floatation performed using zinc sulfate solution with 1.350 specific gravity as previously described [15], revealed barrel-shaped nematode eggs (Figure 3A,B) with overlapping shape and appearance, but slight microscopic differences. The eggs were subjected to a thorough morphological and morphometric analysis based on size and features of polar plugs, wall, and morulae inside the eggs and identified to be *C. aerophila* and *C. boehmi* [1,3,5,23]. In particular, the size of the eggs of *C. aerophila* were within known ranges of 60–65 × 25–40 μm, with morulae filling the eggs and anastomosing ridges and bridges. The eggs of *C. boehmi* also complied with key morphometric features (i.e., 50–60 × 30–35 μm) and presented a space between the morula and the wall and an outer shell presenting fine pitting. Eggs of *C. boehmi* were found also at the microscopic examination of a nasal swab (Figure 3C), performed after the floatation. The Baermann’s examination was negative for nematode larvae, as also the Angio Detect™ test scored negative. 

**Figure 4:**
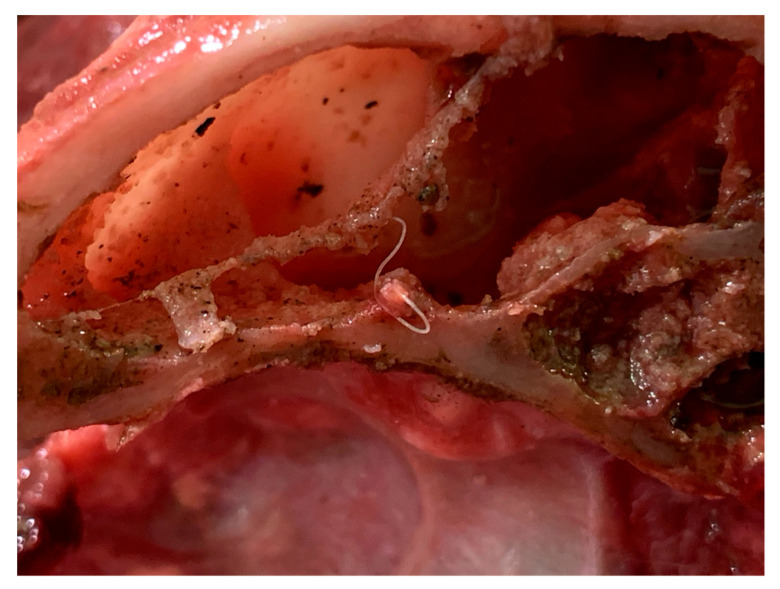
Adult *Capillaria boehmi* retrieved in the paranasal sinuses of the necropsied dog.

**Figure 5:**
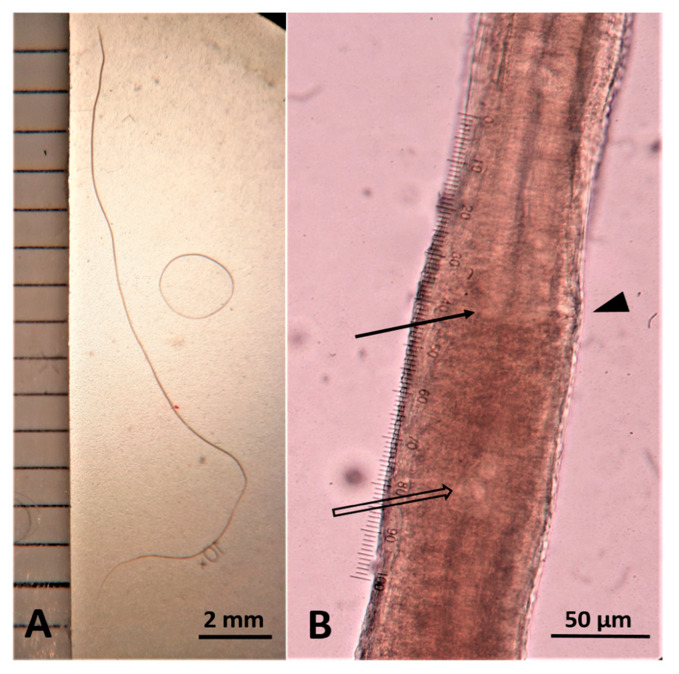
Female *Capillaria boehmi* measuring 30 mm in length (**A**). Junction of the esophagus with the intestine (arrow), vulva (arrowhead), and nucleus of the first stichocyte (empty arrow) (**B**).

At necropsy, three thin and slender nematodes embedded in the mucosa were collected from the nasal sinuses (Figure 4). Two of them were broken during the extraction procedure, while the remaining entire specimen was morphologically examined. It was a female, 30 mm long and 83 μm wide at the level of the end of the esophagus (Figure 5). The esophagus was 5.600 μm long, and the vulva was situated at the level of the junction of the esophagus with the intestine (Figure 5). 

**Figure 6:**
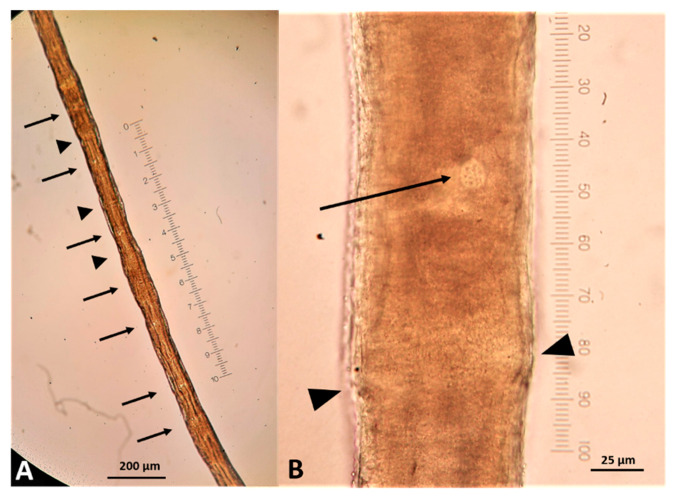
*Capillaria boehmi* stichocytes. Nuclei of stichocytes are visible (arrows). The arrowheads indicate visible contact surface of the cells (**A**). Stichocyte nucleus 15 µm in diameter (arrow) and cell limit (arrowheads) (**B**).

**Figure 7:**
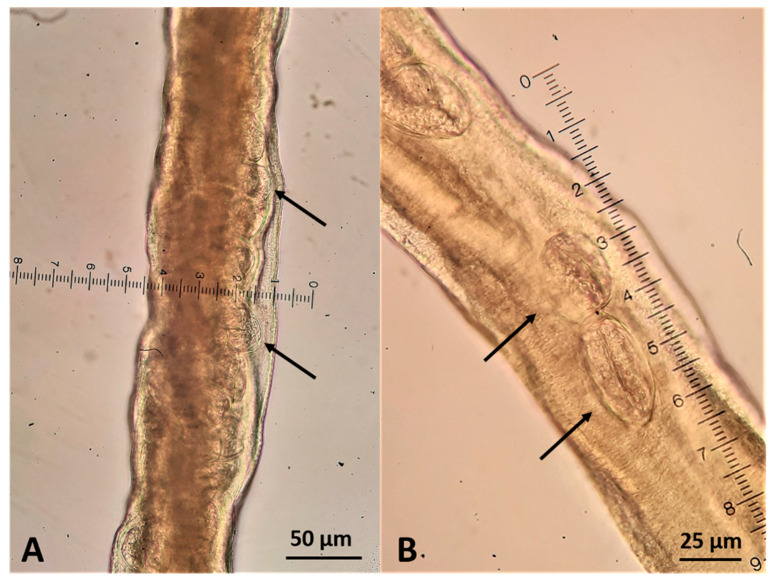
Female *Capillaria boehmi* at the level of the uterus (**A**) that was filled with characteristic barrel shaped eggs (**B**) indicated by arrows.

Twenty stichocytes 200–240 μm long and 72–76 μm wide, with a perceivable nucleus of 15–17 μm diameter at their center (Figure 6), filled the space between the end of the esophagus and the genital pore. The uterus contained eggs whose size was between ~55 μm in length and ~22–23 μm in width, and baring a zygote that was not filling all the egg space (Figure 7). Based on these features, the nematode was identified as an adult female of *C. boehmi*.

### 2.4. Necroscopic Examination and Histopathology

**Figure 8:**
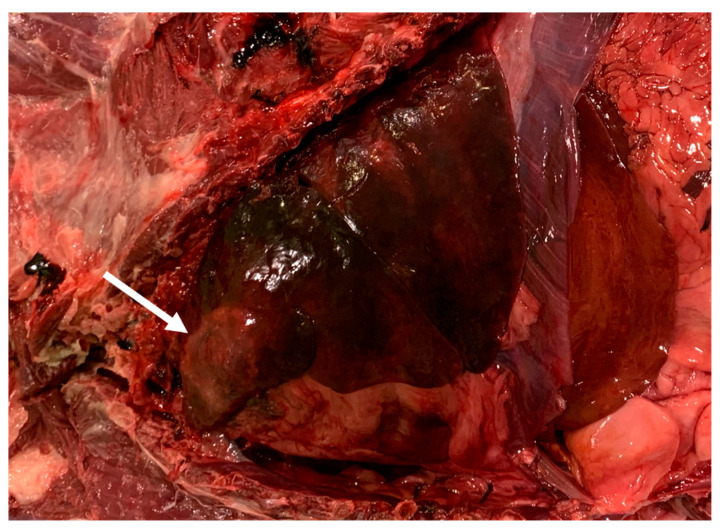
Lung, area of consolidation compatible with respiratory capillariosis.

Catarrhal exudate was present in the lumen of the nasal cavities and of the paranasal sinuses, associated with the presence of white and slender worms (Figure 4). The lungs appeared diffusely congested and edematous and presented multiple areas of consolidation, being the most extended area of about 4 cm in diameter in the left diaphragmatic lobe (Figure 8); visible *C. aerophila* adult specimens were not found. Disseminated, pinhead, and calcified lesions were detected in both kidneys.

**Figure 9:**
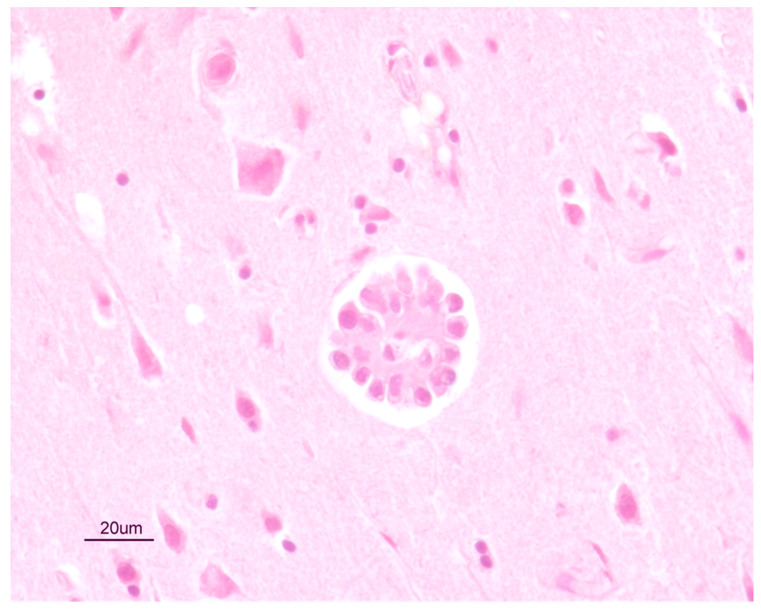
Histological section of a granuloma in the frontal cortex of the brain of the dog, compatible with a parasitic infection.

A wide range of tissue samples, including the brain and the above described gross lesions, were fixed in 10% neutral buffered formalin, embedded in paraffin and routinely processed for histopathological investigations (hematoxylin and eosin stain). Microscopically, pulmonary and renal lesions both consisted of a thick fibrotic capsule, surrounding a central core of necrotic and calcified cellular debris. A severe and diffuse membranous-proliferative glomerulonephritis was also observed, mainly characterized by the marked thickening of the Bowman’s capsule. In the liver, diffuse swelling and vacuolar changes affected the hepatocytes. Scattered perivascular and lymphomonocytic inflammatory foci were observed in the brain, along with a granulomatous lesion in the frontal cortex level (Figure 9). 

### 2.5. Molecular Analysis

Eggs from the floatation and the nasal swab, fragments of the two worms broken at necropsy, and two tissue portions from the olfactory bulbs and the frontal cortex, were subjected to DNA-based assays specific for *C. aerophila* and *C. boehmi* [16,24]. PCR-positive samples were purified using a QIAquick^®^ Gel Extraction Kit (Qiagen, GmbH, Hilden, Germany) and sequenced by a commercial laboratory (BMR—Genomics, Padova, Italy). Sequences were determined in both strands, aligned and then compared with each other and with those available in GenBank using the Basic Local Alignment Search Tool (BLAST; http://www.ncbi.nlm.nih.gov/BLAST). 

The eggs were confirmed to be both *C. aerophila* and *C. boehmi*, and the nematodes were *C. boehmi* specimens. The portions of the brain were also molecularly positive for *C. boehmi*. The sequences obtained for *C. boehmi* and *C. aerophila* were 100% identical to sequences deposited in GenBank under the Accession numbers KR186213.1 and JQ905052.1 respectively [16,24].

## 3. Discussion

This case adds new clinical and epizootiological pieces on current knowledge about respiratory capillarioses of companion animals. 

The clinical scenario was compatible with distinct diseases based on respiratory (i.e., infectious and parasitic pneumonia, neoplasia, cardiogenic edema) and neurological (tumors, poisoning, vascular events, inflammatory/infectious and metabolic diseases) signs. 

Respiratory parasites were included in the first differentials although history, signalment and anamnesis of the dog could have led to an erroneous exclusion of these infections; in fact, the animal had always lived in the garden of a house located in a fishermen town with no relevant movements nor hunting activities which could have put the dog at risk of extra-intestinal parasitoses originating from wild reservoirs. Nevertheless, the combination of lack of routine anthelmintic treatments and compatible respiratory and neurological signs [25,26] led the veterinarian to include canine angiostrongylosis in the differential diagnosis. A floatation assay was included in the laboratory procedures for a comprehensive evaluation of the parasitological status of the dog, along with Baermann’s and antigenic detection tests. The absence of *A. vasorum* fist-stage larvae (L1) and adults in the faeces and pulmonary arteries, respectively, and the negative Angio Detect test indicate that the respiratory disorders were instead caused by the two respiratory capillarioses. This scenario is compatible with the presence of parasitic worms in the airways of the dog, even though such a clinical severity is infrequent in dogs infected by these parasites [3,11,12,13]. 

Pulmonary capillariosis in dogs is often subclinical, as suggested by studies performed in the last decade where some infected dogs, i.e., 9/12 [27] and 13/21 [22], had no apparent signs. The lower respiratory clinical signs and the radiographic findings here described are compatible with pulmonary capillariosis, though the abnormalities detected at X-ray imaging of dogs infected with *C. aerophila* are often non-specific [28]. To date, anatomopathological information on *C. aerophila* infections is scant, probably due to the often subclinical nature of this parasitosis [13]. Additionally, the areas of lung consolidation found at necropsy in the present case are compatible with *C. aerophila*-induced inflammation [13], although alternative disease conditions (i.e., chronic necrotic foci after bacterial infections) cannot be ruled out.

Calcified lesions found in both kidneys were, instead, most probably due to past parasitic migrations, such as those occurring in *Toxocara canis* infections [29].

The hypertrophia and hyperemia of nasal mucosae of the dog here presented were due to the infection by *C. boehmi* [12,13]. Conversely, neurological signs can hardly be put in relation with *Capillaria* spp. in dogs. However, there is a single described case of *C. boehmi*-associated meningoencephalitis, where the infected dog showed generalized convulsive seizures. In this latter case, a cerebral mass was removed via image-guided stereotactic craniotomy and the histopathological diagnosis was severe, locally extensive, meningoencephalitis with a granuloma containing an intra-lesional *C. boehmi* egg. Therefore, an aberrant migration into the cranial cavity of the parasite was the presumed cause of the lesion [20]. In the present case, parasitic stages were not found in the histological analysis of the brain. However, inflammatory foci and granulomatous lesions (Figure 9) in the frontal cortex are compatible with an immunity response to the presence of parasites, in accordance with previous findings [20]. Furthermore, the DNA of *C. boehmi* was found within the olfactory bulbs and prefrontal cortex, further supporting an intracranial migration of the parasite leading to the onset of severe neurological signs. It can be argued that the granuloma found in the present case could have been triggered by *C. boehmi* eggs, other parasitic stages or parasite metabolites, though unidentified. The lack of the detection of parasitic elements in the histological examination of brain sections could be explained by the activation of the local immune response, which is highly specialized in removing foreign bodies or parasite through the phagocytic activity. During their co-evolution process with the host some parasites (e.g., *Taenia solium*, *Toxoplasma gondii*, roundworms) have developed evasion strategies to survive in the central nervous system [30], but it is unlikely that *C. boehmi* modulates or escapes from the inflammatory response as the brain is a rare and an aberrant localization of this nematode. It is also possible that these lesions were caused by a temporary and erroneous migration of the parasites but, if this is the case, it is impossible to determine which stage (e.g., female adults laying eggs or larval stages) migrated through the cribriform plate. The life cycle of *C. boehmi* has not been described in detail thus far and, interestingly, possible endogenous auto re-infections have been suggested based on eggs containing fully developed motile larvae in the upper airways of dogs which were kept infected by *C. boehmi* for years [31]. According to this hypothesis, the eggs could develop to an infective stage while still infecting the host, hatch and re-infect the dog [31]. Although this needs further confirmations, the auto re-infection could contribute to explain the ability of the parasite to persist for years in the host and the records of intracranial localizations of *C. boehmi* as consequences of the direct migration of the larvae through the cribriform plate after their hatching in the upper airways. At present, it is unknown if the tendency of *C. boehmi* to invade the cerebral tissue of dogs could depend on the genetic make-up of the parasite, if brain migrations occur only in cases of heavy parasitic burdens, or if it is a random event. 

Under a clinical standpoint, causes of neurological diseases other than *C. boehmi* infection could be reasonably excluded. The dog had ultrasound findings suggestive of pancreatitis but the clinical scenario was not compatible with a pancreatic encephalopathy, as no typical signs of pancreatitis such as vomit, abdominal pain, dehydration, diarrhea, hypocalcemia, hypo/hyperglycemia, hyperlipidemia, were present [32]. Although described in Pointers, idiopathic granulomatous meningoencephalomyelitis (GME) and pyogranulomatous meningoencephalomyelitis could be also ruled out, as typical pathological features, i.e. lesions widely disseminated in the brain and histologically visible dense aggregates of inflammatory cells surrounding the brain vessels (i.e. perivascular cuffs), were here absent [33]. Mycotic infections were ruled out, as no fungi were evidenced by the Periodic Acid-Schiff (PAS) staining. On the whole, based on (i) the past record published by Clark et al. [20], (ii) the detection of *C. boehmi* DNA in two sites of the brain, (iii) the lack of other noxae explaining the clinical picture, and (iv) the anatomopathological findings, it can be presumed that *C. boehmi* was responsible for the neurological condition in the case here presented. 

The source of infection in the present case is unknown. It is reported that *C. boehmi* may persist for years [31], thus the dog could have become infected during his last travel of the dog in 2016. Foxes are major reservoirs of *Capillaria* spp. affecting companion animals and prevalence is high in this wildlife from northern Italy and bordering countries [6,7,8,9,11]. As the dog was not subjected to routine copromicroscopic examinations nor did it received routine parasiticide treatments, this possibility cannot be ruled out, albeit no history of respiratory distress in the last few years was reported by the owners. It is also plausible that the dog had acquired both infections, even in different time points, due to a possible contamination of the house garden with fox faeces. Accordingly, fox populations are numerous in the region (https://discovermammals.org/what-you-can-do/learn-more/countries/italy/) where the dog originated from (Figure 1).

## 4. Conclusions

Infections by *C. aerophila* and *C. boehmi* are increasingly reported in dogs, especially in those areas where foxes are present [3,7,8,9,13,22,34]. In Italy, *C. aerophila* is enzootic in dogs and cats living in southern regions and, recently, also *C. boehmi* was identified for the first time in dogs from southern Italy, including the region where the dog lived [21,22,24]. Therefore, capillarioses should be included in the differential diagnosis in dogs with respiratory alterations or in the presence of unusual clinical scenarios, e.g. severe respiratory distress, extra-respiratory signs, also in areas where these parasites are unexpected. Respiratory capillarioses can be more severe than believed and more attention should be given in canine clinical practice to *C. aerophila* and *C. boehmi* for several reasons. First, their geographic distribution is appearing in expansion especially as a result of bridging infections from wild reservoirs which are fostered by conurbation to share habitats with domestic animals [13,24]. Second, *C. aerophila* has a zoonotic potential and its eggs can contaminate green public areas [35]: since it may mimic respiratory tumors [2] it is plausible that the incidence of lung capillariosis in humans is overlooked to a certain extent. Finally, nasal capillariosis may cause relevant clinical signs in infected dogs, including partial or total loss of scent and cerebral damages, with an important impact on infected animals [17,31].

In conclusion, respiratory capillarioses should be always included in the differential diagnosis of dogs showing respiratory signs and appropriate copromicroscopic examinations should be performed by clinicians. Veterinary practitioners should be aware of morphological and morphometric features of trichuroid eggs [15] that can be found in dog faeces, to avoid misdiagnoses and set a prompt therapeutic protocol. Moreover, as both *C. aerophila* and *C. boehmi* infections can remain subclinical for a long time, dogs living in endemic areas should be checked routinely for the presence of these respiratory parasites.

## Data Availability

Not applicable.
